# A deep learning approach for morphological feature extraction based on variational auto-encoder: an application to mandible shape

**DOI:** 10.1038/s41540-023-00293-6

**Published:** 2023-07-06

**Authors:** Masato Tsutsumi, Nen Saito, Daisuke Koyabu, Chikara Furusawa

**Affiliations:** 1 Graduate School of Sciences, The University of Tokyo, 7-3-1 Hongo, Tokyo, 113-0033 Japan; 2 Graduate School of Integrated Sciences for Life, Hiroshima University, 1-3-1 Kagamiyama, Higashi-Hiroshima City, Hiroshima 739-8528 Japan; 3 Exploratory Research Center on Life and Living Systems, National Institutes of Natural Sciences, 5-1 Higashiyama, Myodaiji-cho, Okazaki, Aichi 444-8787 Japan; 4 Universal Biology Institute, The University of Tokyo, 7-3-1 Hongo, Tokyo, 113-0033 Japan; 5 Research and Development Center for Precision Medicine, University of Tsukuba, 1-2 Kasuga, Tsukuba, 305-8550 Japan; 6 Jockey Club College of Veterinary Medicine and Life Sciences, City University of Hong Kong, To Yuen Building, Tat Chee Avenue, Kowloon, 999077 Hong Kong; 7 Center for Biosystems Dynamics Research, RIKEN, 6-2-3 Furuedai, Suita, Osaka 565-0874 Japan

**Keywords:** Computational biology and bioinformatics, Systems biology

## Abstract

Shape measurements are crucial for evolutionary and developmental biology; however, they present difficulties in the objective and automatic quantification of arbitrary shapes. Conventional approaches are based on anatomically prominent landmarks, which require manual annotations by experts. Here, we develop a machine-learning approach by presenting morphological regulated variational AutoEncoder (Morpho-VAE), an image-based deep learning framework, to conduct landmark-free shape analysis. The proposed architecture combines the unsupervised and supervised learning models to reduce dimensionality by focusing on morphological features that distinguish data with different labels. We applied the method to primate mandible image data. The extracted morphological features reflected the characteristics of the families to which the organisms belonged, despite the absence of correlation between the extracted morphological features and phylogenetic distance. Furthermore, we demonstrated the reconstruction of missing segments from incomplete images. The proposed method provides a flexible and promising tool for analyzing a wide variety of image data of biological shapes even those with missing segments.

## Introduction

Morphology refers to the biological form and represents one of the most visually recognizable phenotypes across all organisms. Morphological features, including the shapes of organs, tissues, and bodies, are shaped during the developmental process and may evolve over time. Therefore, comparing morphology among species and individuals is expected to provide insight into the functional role of shape and its developmental and evolutionary history^1–5^. To decipher such factors from the morphology, quantification and characterization of shape are critical because it allows us to describe, interpret, and visualize the variations in shape.

So far, a great deal of effort has been made towards shape analysis, and various methods have been proposed. The most widely used shape analysis is landmark-based geometric morphometrics in which landmarks are defined by anatomically homologous points on multiple samples, and the shape of a given sample is characterized by the coordinates of these landmarks^6–10^. The applications of this landmark-based method are wide-ranging, including vertebrates^2,3,11–15^, arthropods^16–19^, mollusks^20,21^, and plants^22,23^. However, there are several difficulties and ambiguities intrinsic to this method despite its prevalence. First, the landmark-based method is unsuitable for comparisons between phylogenetically distant species or distant developmental stages (e.g. between the early and late stages) in which biologically homologous landmarks cannot be defined^10^, while the interspecies comparisons between close species or comparisons among near developmental stages have revealed morphological changes through evolutionary or developmental trajectories^1–5^.

Second, both a large and small number of landmarks can cause the loss of information about the morphology of a sample^8,10,24–26^. In addition, errors can be problematic, such as those from measurement devices^27^ and setting configurations of landmarks set inadequately by researchers owing to differences in skill levels^28^. As the landmark-free method, elliptic Fourier analysis (EFA) has also been proposed^29,30^ and applied to characterize the shape of cells^31,32^, bivalves^33^, fish^11,34,35^, and plant organs^36–38^.

Typically, the landmark-based method or EFA is combined with principal component analysis (PCA) to reduce high-dimensionality in morphological data into easily visualizable low-dimensional space^3,6,11^. Linear methods that reduce dimensionality, such as PCA and linear discriminant analysis (LDA), are straightforward and easily implementable, but a nonlinear approach, such as a deep neural network (DNN), might be suitable for capturing more complex features with fewer dimensions. In fact, nonlinear methods based on DNN have been the standard analysis tools in the fields of image classification^39,40^ and medical diagnostic imaging^41,42^: however, their application to morphological analysis, specifically to feature extraction of morphology, has been still limited to a few cases^43–48^. A possible drawback of the DNN approach is that the analysis is often black-boxed and difficult to interpret, but many attempts have been made to solve this issue^49–51^.

In this paper, a landmark-free method based on a variational autoencoder (VAE) is proposed that analyzes shape from image data without manual landmark annotation. A VAE is a class of DNN and consists of the encoder and decoder. The encoder embeds high-dimensional image data into low-dimensional latent variables, and the decoder reconstructs the input image from the compressed latent variables^52^. The nonlinear-data compressibility of the encoder allows VAE to be used for feature extraction from image data^53,54^. The reconstruction capability of the decoder of VAE ensures that the input image is compressed while maintaining the information of the image, rather than being compressed in an irreversible manner. Herein, the original VAE is modified by integrating a classifier module into the VAE, which allows us to extract morphological features that can best distinguish data with different labeled classes. Although hybrid architectures combining supervised and unsupervised learning have been proposed recently^55–59^, the present study represents the first application of this architecture to morphometrics.

The modified VAE model is demonstrated to be superior to the original VAE and PCA-based methods in capturing morphological features by analyzing the mandibular image data of primates (seven families with a total of 141 samples; see Supplementary Fig. 1e and Supplementary Table 1). The mandible varies widely in morphology depending on its function and diet^60–63^. For instance, the size and morphology of the mandible joint and its position relative to the biting surface differ between carnivorous and herbivorous mammals due to the differences in their masticatory functions^64,65^. The proposed method provides a landmark-free and non-linear feature extraction analysis for the morphological data of a three-dimensional object, as exemplified by the mandible. Additionally, an interpretation of the extracted features is presented as well as the application to the mandibular image data with a missing bone segment. The proposed model is a useful and flexible tool for investigating a morphological dataset.

## Results

**Fig. 1:**
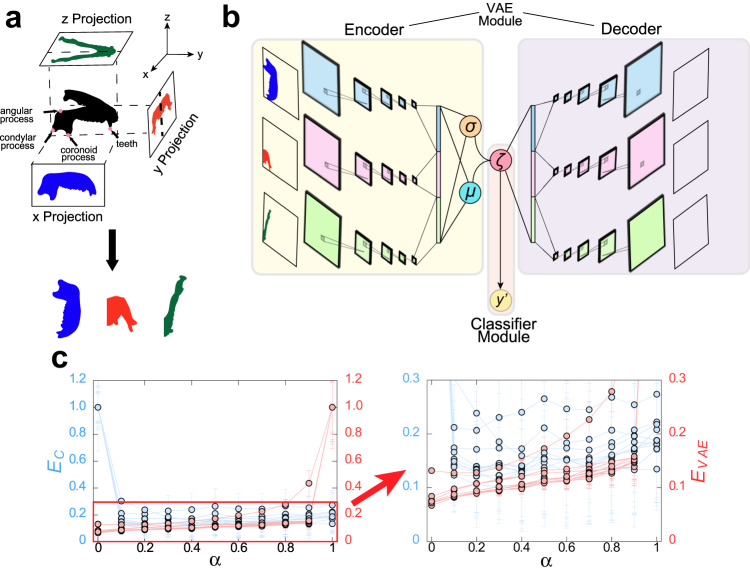
Machine learning pipeline for predicting. **a** Schematic of data preprocessing. **b** Schematic of the Morpho-VAE that comprises the encoder, decoder, and classifier. **c** Plot showing the changes in *E*_*C*_ and *E*_*VAE*_ as *α* is varied: Blue points and red points indicate the values of *E*_*C*_ and *E*_*VAE*_, respectively, in the optimal model for each of the 10 combinations of training and test data. *E*_*C*_ and *E*_*VAE*_ are normalized such that the maximum value is 1. The left panel shows the range from 0 to 1, and the right panel shows the expanded range from 0 to 0.3.

The study aims to develop a landmark-free method for extracting morphological features from images to distinguish different groups. A total of 147 mandibles samples from seven different families (i.e., seven labels) were prepared for verifying the method. These samples comprise 141 samples of the primate mandibles (Cercopethecidae, Cebidae, Lemuridae, Atelidae, Hylobatidae, and Hominidae) and six samples of the mandibles of carnivora (Phocidae) as an outgroup. Here, Phocidae samples were added to examine whether or not the proposed method can distinguish data with apparently different morphology. The corresponding three-dimensional mandible data are projected from three directions to produce three projected two-dimensional images, as shown in Fig. 1a (see “Methods” section). These three projections of each mandible are used as the input images for the following analysis. The proposed architecture, morphological regulated variational auto encoder (Morpho-VAE), is illustrated in Fig. 1b. Note that the VAE module is combined with the classifier module through the latent variable *ζ*. Since we aim to extract features that can classify families while maintaining the quality of reconstruction by VAE, we constructed a total loss function *E*_*total*_ = (1 − *α*)*E*_*VAE*_ + *αE*_*C*_, as a weighted sum of the VAE loss (*E*_*VAE*_) and the classification loss (*E*_*C*_). *E*_*VAE*_ is the loss associated with VAE (i.e., the reconstruction + regularization losses), *E*_*C*_ is the classification loss for the classifier module, and *α* is a hyperparameter that dictates the ratio between *E*_*VAE*_ and *E*_*C*_ in *E*_*total*_. Using the mandible sample images, the hyperparameter *α* is determined as 0.1 through cross-validation (Fig. 1c, see also “Methods” section). This choice of *α* ensures a low *E*_*C*_ with a negligible increase in *E*_*VAE*_ from *α* = 0, indicating that the classification ability can be incorporated into the VAE without lowering the performance in the VAE module. Other hyperparameters, such as the number of layers, number of filters, type of activation function, and optimization function, are also tuned; moreover, the number of dimensions of the latent variable are set to three (see “Methods” section).

### Cluster separation

After the 100-epoch training, as described in the “Methods” section, a trained model is obtained that can classify the input image into seven class labels with a high validation accuracy (90% as median, Supplementary Fig. 2b), compress the image into three-dimensional latent space *ζ*, and reconstruct the image from the latent space.

**Fig. 2:**
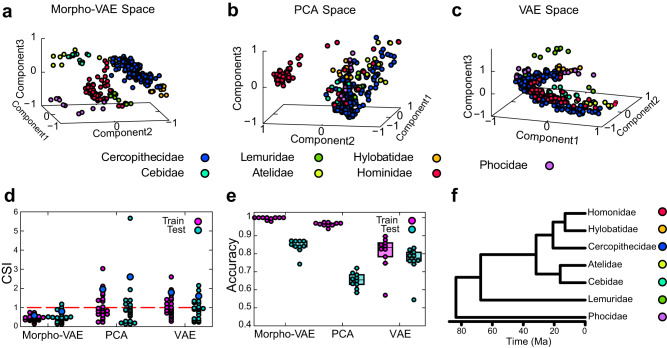
Distribution of data in latent space. **a**–**c** Data distribution in latent space: By using Morpho-VAE, PCA, and VAE, all the input images are the same and are dimensionally compressed into a three-dimensional latent space by each of the methods. **d** Dot plot of CSI; a point below 1 represents a pair of well-separated clusters: Blue dots represent Davies--Bouldin indices for different models. **e** The boxplot of classification accuracy of families by SVM as a measure of cluster separation. We adopted the SVM with the radial-basis-function kernel. The regularization parameter is 1.0, the tolerance for the stopping criterion is 0.001, and the coefficient of kernel is 1/3 (i.e., 1/latent dimension). Each point represents the classification accuracy of the 10 tuned models. The boxplot shows the median and the quartile range of the data. Morpho-VAE shows a trend toward higher classification accuracy than PCA and VAE (Steel test Morpho-VAE - PCA *p* = 3.04 × 10^−4^, and Morpho-VAE - VAE *p* = 4.70 × 10^−3^ for test data). **f** Phylogenetic tree that was created by VertLife.org (http://vertlife.org/phylosubsets/)^77^. The family-level phylogenetic tree was created by selecting the species with the largest sample size for each family.

The distribution of training and validation datasets in the latent space (Fig. 2a) illustrates that the data points of each label form well-separated clusters from the data with different labels. Here, to confirm that the label information can separate the clusters, the latent space distribution in Morpho-VAE is compared to that in PCA (Fig. 2b) and VAE (Fig. 2c), showing that the clusters are most separated in Morpho-VAE space (Fig. 2a). Herein, PCA is performed by transforming the image into a vector of 16,384 (= 128 × 128) dimensions and extracting the top three components. Note that this use of PCA differs from its ordinary use in the landmark method^6,7,10^ and the elliptic Fourier analysis^32,66^, where not a vector of pixel data but the coordinates of landmarks or Fourier coefficients are subjected to PCA. VAE is trained using the same procedure and training, validation, and testing datasets to Morpho-VAE, as described in the Methods section, while ignoring classification loss (i.e., *α* = 0). To quantify the extent to which the data points with different class labels are separated in each method, the cluster separation index (CSI) is defined as follows:

1 $${{{{\rm{CSI}}}}}_{ij}=\left(\frac{{\delta }_{i}+{\delta }_{j}}{{\Delta }_{ij}}\right),$$

where ${\Delta }_{ij}=\parallel {{{{\bf{x}}}}}_{G}^{i}-{{{{\bf{x}}}}}_{G}^{j}{\parallel }_{2}$ is the Euclidean distance between the centroids of the *i*-th cluster *C*_*i*_, ${{{{\bf{x}}}}}_{G}^{i}$, and the *j*-th cluster *C*_*j*_, ${{{{\bf{x}}}}}_{G}^{j}$. ${\delta }_{i}=\sqrt{1/| {C}_{i}| {\sum }_{k\in {C}_{i}}\parallel {{{{\bf{x}}}}}_{k}^{i}-{{{{\bf{x}}}}}_{G}^{i}{\parallel }_{2}^{2}}$ is the mean distance between a point in *C*_*i*_, ${{{{\bf{x}}}}}_{k}^{i}$, and the *i*-th cluster centroid, ${{{{\bf{x}}}}}_{G}^{i}$. When the clusters *i* and *j* are separated, CSI_*ij*_ < 1, and CSI_*ij*_ > 1 when one of the clusters is encompassed or partially overlaps the other one. By taking the average of the maximum of CSI_*ij*_ for *j* ≠ *i* (i.e., $\mathop{\sum }\nolimits_{i = 1}^{7}{\max }_{j\ne i}{{{{\rm{CSI}}}}}_{ij}/7$), this index corresponds to the Davies–Bouldin index with *p* = *q* = 2 ^67^, which is widely used to evaluate the degree of cluster separation. Figure 2d shows the CSIs for all pairs of the seven clusters obtained in the reduced feature space of Morpho-VAE, PCA, and VAE, in which a single circle indicates a pair of different classes. In Morpho-VAE, almost all points are less than one, which indicates that all pairs of clusters are well-separated; however, for PCA and VAE, almost half of all points are lower than one, suggesting that the data points with different family labels cannot be distinguished in PCA or VAE space. For further verification, the evaluated Davies–Bouldin indices (a score of less than 1 represents well-separated clusters) are 0.80 (Morpho-VAE), 2.60 (PCA), and 1.60 (VAE) for test data.

Additionally, the classification accuracy calculated using the support vector machine (SVM) from the data distribution in the latent space is quantified as another measure of the degree of cluster separation. Because the SVM can solve a classification problem with a high validation accuracy when the clusters of data with different labels are well-separated in the latent space, this SVM-based accuracy is expected to reflect the degree of cluster separation. After the proposed Morpho-VAE is trained using the training data (for PCA, the top three PC vectors from the training data are selected), the same training data are used for training the SVM, and then the SVM accuracy in the latent space is calculated using the test data. The average test accuracy estimated from 10 different combinations of training and test data is shown in Fig. 2e. We performed a Steel test^68^ to determine if Morpho-VAE and PCA, as well as Morpho-VAE and VAE, differed in their classification accuracy. Morpho-VAE model achieves a considerably higher test accuracy than PCA and VAE (*p* = 3.07 × 10^−4^, *p* = 3.70 × 10^−3^ (Fig. 2e)), indicating that the proposed model can embed the data of different families in well-separated clusters in latent space.

Since Morpho-VAE is the only method that utilizes supervised information about families for training, the higher clustering performance of Morpho-VAE shown in Fig. 2 does not necessarily indicate that Morpho-VAE is inherently superior to the other two methods that do not use supervised information. However, the results above demonstrate that Morpho-VAE is capable of generating a suitable latent space for effectively separating different morphologies by integrating VAE with supervised information. Additionally, we conducted an evaluation to determine whether the clustering performance of the latent space generated by Morpho-VAE is superior to that of PCA and VAE, regardless of the use of supervised information. Our hypothesis is that the latent space of Morpho-VAE, designed to separate mandible morphologies of different families, can effectively cluster a morphology dataset from an additional family that was not included in the training process. To test this hypothesis, we performed the following analysis: First, we trained Morpho-VAE using the training dataset of six families out of the seven families prepared, utilizing family information to generate a latent space suitable for separating the morphologies of these six families. Next, we calculated the CSI between the additional family dataset and the test datasets of each of the six families on the latent space of Morpho-VAE. Similarly, for PCA and VAE, we constructed latent spaces using datasets of six families and calculated the CSI for the additional family dataset. The maximum value of CSI between the additional family dataset and each of the pre-existing six families was used as the measure of clustering performance for the newly added family dataset. It is important to note that we did not use family label information in evaluating the clustering performance of the additional datasets, allowing us to make a fair comparison of the clustering performance among Morpho-VAE, PCA, and VAE. Supplementary Fig. 8 presents the results of our analysis. For example, Supplementary Fig. 8f displays the maximum CSI between Hominidae and six other families in the latent space generated without the Hominidae dataset. As shown in Supplementary Fig. 8, Morpho-VAE resulted in lower maximum SCI scores (indicating better cluster separation) compared to PCA and VAE for the majority of cases. This result suggests that Morpho-VAE is capable of generating a better latent space for separating mandible morphology compared to PCA and VAE. This superior performance of Morpho-VAE may be attributed to the fact that Morpho-VAE tends to focus on informative segments of images to characterize mandible morphology, allowing for separation of morphologies of different families even without label information. The data distribution in the latent space (Fig. 2a) shows that the distances between clusters are different for each pair of clusters. This distance in the latent space can be interpreted as the similarity of shapes. In terms of classification, Hylobatidae and Cebidae are easy to distinguish, but Atelidae and Cercopithecidae are difficult to distinguish, and so on (Supplementary Fig. 1e). This shape similarity may be hypothesized to be determined based on evolutionary distance; however, the relationship between morphological similarity and evolutionary distance has long been a topic of debate^69–76^. This is because other factors, such as diet (carnivore, herbivore, or omnivore), sexual dimorphism, and predator presence, may have a greater influence on morphology than evolutionary distance. To assess whether our mandibular data support this hypothesis, we investigated the correlation between latent spatial distance and phylogenetic distance across families. For this family-level comparison, we selected a representative species with the largest sample size from each family data and generated a family-level phylogenetic tree from VertLife.org (http://vertlife.org/phylosubsets/)^77^. We selected *Macacafuscata* (as Cercopithecidae), *Cebuscapucinus* (Cebidae), *Lemurcatta* (Lemuridae), *Atelespaniscus* (Atelidae), *Hylobateslar* (Hylobatidae), *Homosapiens* (Hominidae), and *Zalophuscalifornianus* (Phocidae). The generated family-level phylegenetic tree is shown in Fig. 2f. The result of the comparison is illustrated in Supplementary Fig. 3, where no correlation is observed between the distance of clusters in the latent space and the family-level phylogenetic-tree distance.

### Reconstructing and generating images from latent space

**Fig. 3:**
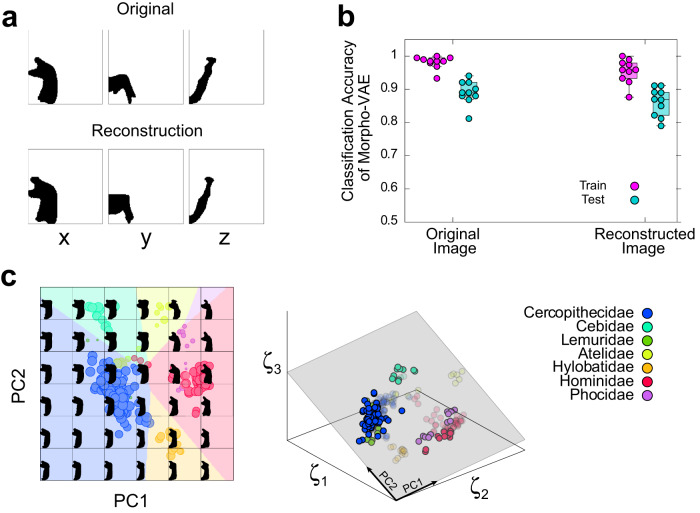
Image reconstruction by the proposed Morpho-VAE. **a** Comparison between the original and reconstructed images. **b** Classification accuracy of the reconstructed images: The boxplot of the classification accuracy of the original and reconstructed images using the classification module. Each point represents the classification accuracy of the 10 tuned models. Statistically significant differences in distribution between the reconstructed and original images were not identified (Mann--Whitney *U*-test: *p* = 0.160 for test). **c** Generating images from latent space: The left figure shows the images reconstructed from the grid points in the PC plane at PC3 = 0 in the Morpho-VAE space, and each point is a data point projected from the Morpho-VAE space onto the PC plane. The size of each point is proportional to the absolute value of its distance from the PC3 = 0 plane. The larger the size, the closer the point is to the PC3 = 0 plane. The right figure shows the positions of points with regards to the PC plane (gray colored). The contribution ratios of PC1 and PC2 are 51.2% and 28.1%, respectively.

The proposed Morpho-VAE model can reconstruct an image from the low-dimensional latent variable *ζ* through the decoder as well as compress the input image into *ζ* through the encoder. This ability guarantees that the compressed latent variable *ζ* preserves the information about the morphology of the input data, rather than compressing them in an irreversible manner. A representative example of an input and reconstructed images from the input image is shown in Fig. 3a, in which the entire morphological information of the input image is preserved in the reconstructed image, and some detailed differences are recognizable. The reconstruction loss *E*_*Rec*_ that reflects the accuracy of the reconstructed input image reaches a plateau during training (Supplementary Fig. 2c), indicating that learning is successful. The reconstructed image is re-input into Morpho-VAE to further confirm the extent of morphological information preserved in the reconstruction image; subsequently, the predicted label is obtained through the classifier module and the prediction accuracy is calculated by comparing with the true label. This prediction accuracy can be used as an indicator of the extent of morphological information that is preserved as the precisely reconstructed images should be correctly classified, but the poorly reconstructed images should result in a significant accuracy drop. Figure 3b illustrates this prediction accuracy of the reconstructed image in comparison to the accuracy calculated from the original data with only a few percent of drops observed. We further confirmed that there was no significant difference between these accuracies by performing a Mann–Whitney test (*p* = 0.160). This suggests that the reconstruction is demonstrably successful.

Similar to VAE, the Morpho-VAE model is categorized as a class of generative models that can generate an image from an arbitrary point in the latent space *ζ* even when no input data correspond to the point in *ζ*. This property enables the visualization of the latent space; Fig. 3c illustrates the generated images from the uniformly sampled *ζ* on the two-dimensional square lattice in three dimensional latent space (right panel in Fig. 3c) in which the choice of the two dimensional plane in the three-dimensional latent space is determined by PCA based on the data distribution in the latent space. The background colors in the left panel of Fig. 3c represent the predicted labels from *ζ* by the classifier module; circles indicate the input data points mapped into *ζ* with their sizes corresponding to the distance from the PC1–PC2 plane. The generated morphology changes gradually in the latent space (left panel in Fig. 3c), indicating that a smooth embedding is achieved of the morphological information into the latent space. In addition, both PC1 and PC2 seem to reflect an anatomical meaningful feature because the angle between the condylar and the coronoid processes approaches 90 degrees as PC1 becomes larger (left panel of Fig. 3c), and the angular process becomes larger as PC2 increases.

### Visual explanation of the basis for class decisions

**Fig. 4:**
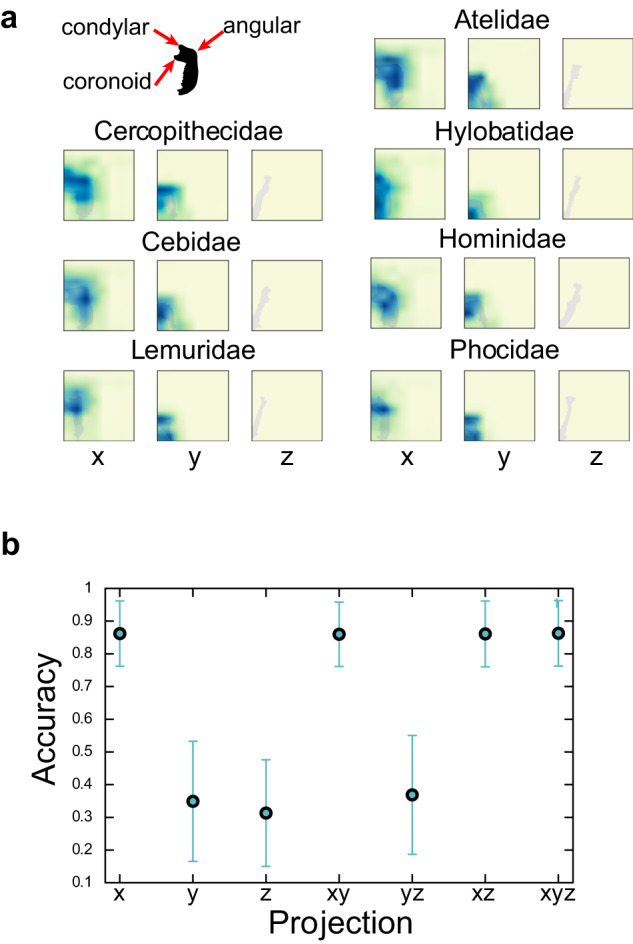
Visualization of the saliency map by Score-CAM. **a** Saliency map in each family calculated by the Score-CAM method: The stronger the color, the more intensively the area is highlighted for classification. **b** The horizontal axis is the projection direction used for the input image (e.g., *xy* indicates that the input image in the *z* direction is a blank image). The vertical axis refers to the class classification accuracy using the input. The error bars indicate the mean and standard deviations in the accuracy for each of the 10 tuned models.

The part of the image that Morpho-VAE focuses on in the classification task can be interpreted. Herein, a post hoc visual explanation method Score-CAM^51^ is used for visualizing important areas in the input image for classification. The schematic overview of Score-CAM is given in Supplementary Fig. 4 (see “Methods” section for detailed procedures). Outcomes of this analysis are “the saliency maps” for each family, as shown in Fig. 4a in which the darker colors represent the area judged more important for classification by the Morpho-VAE. These maps emphasize essential bone processes: the area around the coronoid process (Fig. 1a) for Phocidae, the condylar process for Cercopethecidae, Hylobatidae, and Hominidae. Furthermore, the angular processes, except for Hylobatidae, are highlighted in the *x* and *y* projections. These processes connect temporal and pterygoid muscles as well as are crucial in the opening and closing of the jaw; therefore, them being highlighted for classification is reasonable.

The Score-CAM analysis also clarifies that the images of *z* projection do not contribute to the classification task as the colormaps in *z* projection are all blank (Fig. 4a). This result is further confirmed by calculating the classification accuracy from the inputs of single-direction data only (e.g., *x* projection only) and those of double-direction data only (e.g., *x* and *y* projections only), rather than the full dataset of *x*, *y*, and *z* projections (Fig. 4b). Both results indicate that the *x* projection image is most informative. Likewise, the site around the teeth in the *x* projection (bottom half of the image) tends to be ignored by the map, which likely reflects that the position of the teeth and their presence/absence varies greatly among samples and is thus less informative.

### Reconstruction from cropped data

**Fig. 5:**
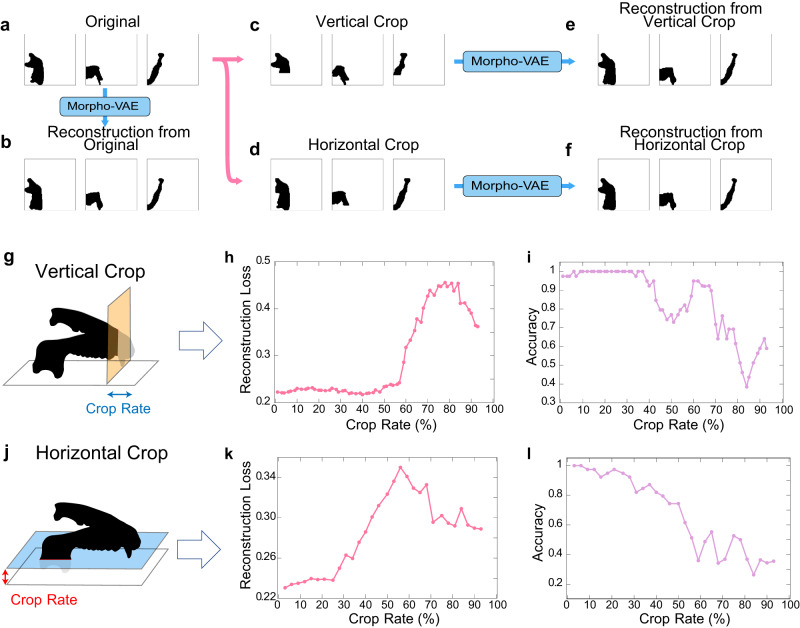
Reconstruction of cropped image. **a**–**f** Procedure of image cropping and reconstruction: the figures are the mandibles of *Homo* *sapiens*. **c**, **d** represent 40% vertical and 32% horizontal cropping, respectively. **g**–**l** Reconstruction loss and accuracy after vertical and horizontal image cropping: Crop rate is the percentage of the mandible missing relative to its vertical or horizontal length. The figures in the first column exemplify the cropping of the mandible data. The graph in the second column shows the reconstruction loss between the reconstructed and original images. The graph in the third column shows the classification accuracy of the reconstructed images.

Bone samples, especially fossil samples, sometimes have missing parts. A possible application of the generative ability of the proposed model is to reconstruct such missing bone parts based on the remaining parts. Herein, the proposed model is demonstrated to achieve this reconstruction from a partially cropped image. Artificially cropped three-dimensional data from the *y* and *z* directions (Fig. 5g, j) are prepared and their *x*, *y*, and *z* projections are used as the data set to be reconstructed. Figure 5a, c, d show representative examples of the original, vertically cropped, and horizontally cropped data, respectively, and their reconstructions using the proposed Morpho-VAE are presented in Fig. 5e (vertical crop) and Fig. 5f (horizontal crop). The reconstructed images from the cropped data (Fig. 5c, d) illustrate that the cropped area in the mandible of the original image (Fig. 5a) is reconstructed well but not perfectly. The image looks closely similar to the reconstructed image from the original (Fig. 5b), indicating that the cropped region is less informative than the remaining region.

Furthermore, the robustness of this reconstruction is evaluated by calculating the cropped-region dependency of the reconstruction loss, i.e., the binary cross-entropy between the reconstructed image from the cropped data and the original image (Fig. 5h, k, respectively) as well as that of the prediction accuracy (Fig. 5i, l). Within about 60% and 25% crop rates for the vertical (Fig. 5h, i) and horizontal (Fig. 5k, l) crops, respectively, only a slight increase in the loss and drop in the accuracy is observed, indicating that the reconstruction quality is maintained. The loss then starts to increase and the accuracy drops for a further increase in the crop size. For the vertical crop, an image with the cropping size just before the loss starts to increase is shown in Fig. 5d in which the shapes of the coronoid and condylar processes are just barely preserved. When these processes are completely removed, the reconstruction and classification fail (Supplementary Fig. 5). For the horizontal crop, an image just before the loss increase (Fig. 5c) shows that the reconstruction is robust against the cropping of the region around the teeth and tip region of the mandible (i.e., the region around the body of the mandible). Both the aforementioned results indicate that the shape of the coronoid and condylar processes contain relevant information about the overall shape of the mandible, which is consistent with the results of the Score-CAM analysis (Fig. 4a).

## Discussion

In this study, a method based on VAE combined with a classifier module is proposed for morphological feature extraction and analyzing the image datasets of mandibles. The proposed method compresses the 128 × 128 pixel input image data into three-dimensional latent space in which the data points of different families form well-separated clusters and the degree of cluster separation outperforms those obtained using the unsupervised dimension-reduction methods, i.e., VAE and PCA (Fig. 2 and Supplementary Fig. 8). Because the label information of image data is used as the supervisory signal for the classifier module, the proposed model incorporates the essence of supervised learning as well as that of unsupervised learning of a VAE module. This architecture is designed to reduce dimensionality by focusing on the morphological features through which the differences between predefined labels (i.e., family classes) are distinguished. Consequently, the proposed Morpho-VAE can be interpreted as a nonlinear version of LDA that is designed to determine a linear combination of features that separates data with different classes.

While hybrid architectures of Variational Autoencoder (VAE)-based unsupervised learning and classifier module have been investigated for solving classification tasks with limited labeled data and a large number of non-labeled data^78,79^, their application to dimensionality reduction and feature extraction has been studied more recently. For example, Bandyopadhyay et al.^56^ utilized this architecture to extract features from drawings by dementia patients to distinguish between dementia and non-dementia cases. Similar architectures have been extended to handle multimodal inputs for anomaly detection in robotic vehicles under uncertain environments^55^, or for classification of diverse cancer types using omics data^57^. Furthermore, this hybrid architecture has been proposed to be combined with a loss function that ensures equally spaced clusters with each label in the latent space, resulting in high-performance classification and reconstruction^59^. Building upon these previous studies, the present study provides the first application of this architecture to morphometrics and presents a framework for landmark-free morphological quantifications.

The results in Fig. 1c also indicate that the reconstruction loss exhibits negligible increase after taking into account the classification loss, as depicted in Fig. 1c with *α* = 0 (reconstruction only) and *α* = 0.1 (reconstruction and classification), suggesting that the reconstruction performance can be maintained to some extent by adding the classification function; moreover, this ensures the cluster separation of different-label data in the latent space. A supervised dimensionality-reduction technique such as between-group PCA(bgPCA) can cause spurious separation^80–82^ for a small sample size. To avoid this, we performed the cross-validation procedures by separating data into training, validation, and test data, which corresponds to the operation performed by Cardini and Polly^82^. With the use of CNNs, this procedure successfully avoided overfitting and distinguished seven family groups with high test accuracy, even for a small sample size.

The characteristics of this model, which select the latent space that distinguishes predefined labels, can be described as extracting morphological features by focusing on traits through which a clade is well distinguished from others. The distance in the latent space is then considered to be a measure that contains information about these traits. Although we examined whether or not there is some link between this distinguishability and the evolutionary distance, no clear correlation between the latent-space and phylogenetic distances was detected (Supplementary Fig. 3). As was seen in our result, the longstanding debate regarding the correlation between phylogenetic and morphological distances has been extensively discussed, as the relationship is not always straightforward^69–76^. Several studies have successfully demonstrated that phylogenetic relationships can be inferred from morphological differences. For instance, recent studies utilizing deep learning approaches with embedding techniques, such as the “triplet loss” method, have shown promising results in phylogenetic reconstruction using images of butterflies^43^ and rove beetles^83^. However, these studies were mostly limited to comparisons among closely related taxa. This is because the presence of homoplasy, including reversal, parallel, and convergent evolution^72^, can confound morphology-based estimation of phylogenetic relationships. These difficulties would manifest in comparisons among wider taxa. One possible reason for the lack of a significant correlation between latent state distance and phylogenetic distance in our study may be due to the inclusion of phylogenetically broad and distant taxa, coupled with a relatively small dataset size. Furthermore, previous studies have suggested that mandible morphology can exhibit a significant degree of non-genetic variance and homoplasy^84^ resulting from adaptations to dietary habits. These factors can further complicate the observation of the relationship between phylogeny and morphology. Therefore, we speculate that the absence of correlation between latent space distance and phylogenetic distance does not necessarily indicate limitations in our proposed method. To address this, future work will involve verifying the applicability of our method using morphological data from more closely related taxa, possibly by combining the latest advanced embedding technique using the triplet loss function^43^.

Another potential explanation for the lack of correlation between morphological and phylogenetic differences is the presence of other systematic morphological differences that disturb the correlation. To explore this possibility, we investigated whether sex differences could be identified in the latent space of Morpho-VAE (Supplementary Fig. 6). However, our findings did not reveal any evidence of sex differences, indicating that sex did not significantly contribute to the correlation analysis of morphology and phylogeny. In contrast to a previous study that detected sex and age differences in the human mandible^85^, our study employed size normalization, utilized mixed data from multiple families, and conducted supervised classification of these families. This size normalization was not adequate to detect sex differences and may have obscured the features that distinguish between the sexes. We conducted this analysis for exploring factors that would disturb the correlation between morphological and phylogenetic differences, however, if the main aim is to detect the sex difference, the analysis without the size normalization would be required. Additionally, the limited sample size for certain families presented a significant challenge in our analysis. These unsuccessful results suggest that a narrower taxonomic comparison should have been employed if the focus was on detecting correlations between phylogeny, morphology, or sex differences.

In this study, we used data that were apparently different and not difficult to classify from anatomical viewpoints to validate the usefulness of the proposed landmark-free method. To further check the application to more, morphologically similar data, we examined the genus-level comparison on a family dataset, namely, whether Cercopithecidae dataset can be divided into four genera, Cercopithecus, Macaca, Mandrillus, and Papio. The number of data was 20 for Cercopithecus, 92 for Macaca, 10 for Mandrillus, and 16 for Papio. Supplementary Fig. 7e–g show the results of the comparison after the hyperparameter tuning and training with four genera labels. The distribution of the four genera output by Morpho-VAE were well separated compared with PCA and VAE. We computed CSI and classification accuracy using SVM to measure the degree of separation of the clusters’ output using Morpho-VAE, PCA, and VAE. Supplementary Fig. 7h, i illustrate that data points with different labels in Morpho-VAE were still more separated than those in the other methods. These results show the applicability of the proposed method to the genus level data as well.

Furthermore, the Score-CAM method, which provides an interpretable visualization of the parts of an image that are important for classification (Fig. 4), was applied to overcome the difficulty of interpreting DNN-based analysis. The first notable result of this analysis is that the *x* projection of the mandible image data is the most important for classification among the *x*, *y*, and *z* projections. This result is likely attributed to the fact that the area of the *x* projection is the largest and the results of Score-CAM, which focuses on the lateral view of the mandible is consistent with the previous studies in which the landmarks visible from the lateral view of the mandible are important for detecting sexual dimorphism^86–88^ and inter-period variation^89^. Moreover, the analysis through a closer look at the *x* projection shows that the anatomically distinguishable projections of bone, i.e., the angular, condylar, and coronoid processes, are highlighted. For all groups except for Hylobatidae, the angular process is highlighted, but the condylar process for Cercopithecidae, Hylobatidae, and Hominidae are exaggerated. The angular and coronoid processes provide insertion sites for the medial pterygoid and temporalis, respectively; both of which are critical for producing bite force^65^. The coronoid process provides the temporomandibular joint, which works as the fulcrum during biting. The highlighted parts essentially correspond to key regions related to mastication; thus, them being highlighted seems reasonable. For Phocidae, the area around the coronoid process is emphasized. This is reasonable because a well-developed temporalis is a key feature of carnivora, and the coronoid process to which the temporalis inserts is notably enlarged compared with the other two processes.

As an application of the generative aspect of the model, the proposed model is demonstrated to complement a missing bone segment from an artificially cropped image (Fig. 5) based on the remaining structure. The reconstruction is robust against the cropping of the region around teeth and tip of the mandible (Fig. 5c, h, i), but sensitive to the lack of the mandibular joint, i.e., the coronoid and condylar processes (Fig. 5d, k, l). Both these results are consistent with the results of the Score-CAM analysis (Fig. 4a) in which the shape of the bone processes contains relevant information about the overall shape of the mandible. The proposed model can reconstruct a missing segment from data having defects, i.e., data in which a part of the sample is missing or damaged, as is often the case with fossils. Although there exist landmark-based methods that can interpolate missing landmark locations^90^, the proposed model has the flexibility of reconstructing the entire missing segment from the remaining structure. The generative model based on VAE has also been applied to jaw reconstructive surgeries for completing the missing segments of the bone based on the remaining healthy structure^91^. The proposed architecture, by combining a VAE and classifier module, provides a new framework for reconstructing missing bone segments while performing dimensional reduction for visualization and classification.

In summary, the proposed model enables dimension reduction and feature extraction by which different label data are well-separated, providing a promising application of analyzing morphological dataset in biology. A comparison of the proposed method with landmark methods needs to be performed in the future, but even if the performance of the method is comparable to the conventional methods, the proposed landmark-free method provides a useful tool to non-experts, without need for manually defining the landmarks. Although the model is designed for image input data, a combination with the landmark-based method is possible, for instance, the model output through Score-CAM analysis (Fig. 4) can be used for defining the landmark positions in a systematic manner. In addition, the proposed model can be modified in the future to extend to three-dimensional input data, which will provide a deeper analysis and higher resolution of the reconstructed image, but that will also require a high machine power and a huge dataset.

## Methods

### Data sets and data preprocessing

Three-dimensional computed tomography (CT) scanning morphological data of primate mandibles were collected from Primate Research Institute (KUPRI) and MorphoSource.org. Phocidae (the carnivores) was used as an outgroup to highlight the difference between herbivores and omnivores. Additionally, three-dimensional datasets were collected, which consist of three images of the mandible captured from three orthogonal directions (i.e., top-, front-, and side-views), from Mammalian Crania Photographic Archive Second Edition (MCPA2). A total of 148 mandible datasets (87 Cercopethecidae, 6 Cebidae, 6 Lemuridae, 6 Hylobatidae, 6 Atelidae, 30 Homonidae, and 6 Phocidae) were collected (Supplementary Table 1). Samples were restricted to full adults with no abnormalities in appearance.

Because deep learning using three-dimensional data requires extensive computational resources and large memory size, accompanied by the memory-access problems^92–94^, here, we converted three-dimensional mandible-image data into three two-dimensional images (i.e., top-, front-, and side-views) to avoid these challenges. Supplementary Fig. 1a illustrates that the mandible is aligned such that its teeth face downward, and the *xy* plane is defined as the plane to which the base of the mandible is parallel. Next, the position of the mandible is adjusted such that the line connecting the center of the two medial tips of the condylar head and the mandible tip is parallel to the *y*-axis. Because the mandibles of all the animals collected in this study are left–right symmetrical, one mandible is divided into two pieces by the center of the mandible tip to increase the number of datasets; moreover, one part is mirror-image inverted. The divided mandible, which is placed in the *xyz* space, is then converted into a set of three two-dimensional images with a size of 128 × 128 pixels by projection onto the *yz* (*x* projection), *xz* (*y* projection), and *xy* (*z* projection) planes. In addition, the samples we analyzed are roughly five times different in size. Without the size normalization, the smaller images would be distorted unless the input images are of high resolution. To avoid size dependency of data, we downsized the projected images so that the length from the angular process to the tip of the mandible is normalized to be the same (Supplementary Fig. 1d).

### Model description

This study aims to extract low-dimensional image features while ensuring the ability to classify the mandible images into families. To this end, Morpho-VAE (Fig. 1b), a VAE-based model, is proposed in which a VAE module is combined with a classifier module through the latent variable *ζ*. Similar to the conventional VAE, the VAE module of the Morpho-VAE model comprises a *l*-layer convolution neural network as the encoder and a *l*-layer deconvolution neural network as the decoder. The encoder is a layer for reducing the input data into a low-dimensional latent variable *ζ* in which the input image is converted into the mean *μ* and variance *σ* of the multidimensional normal distribution. Subsequently, the latent variable *ζ* is sampled from the distribution ${{{\mathcal{N}}}}(\mu ,\sigma )$. The decoder is a layer for reconstructing the low-dimensional latent variable *ζ* into an output image that has the same resolution as the input image. The network is trained such that the output image is as close as possible to the input data by optimizing the reconstruction loss *E*_*Rec*_ (see below). The distinct feature of Morpho-VAE is that the VAE module is combined with a classifier module in which a single-layer network converts the low-dimensional latent variable *ζ* into the output vector for classification using the softmax activation function (Fig. 1b). Therefore, Morpho-VAE has two outputs: the output image for the reconstruction and the output vector for the classification. The classifier module is trained to predict the label from the input data via the latent variable *ζ* in a supervised-learning manner. Herein, family-level classification from the input image is considered; therefore, the training labels are: Cercopethecidae, Homonidae, Cebidae, Lemuridae, Hylobatidae, Phocidae, and Atelidae. A more detailed architecture of Morpho-VAE is shown in Supplementary Fig. 2e.

The loss functions *E*_*total*_ required to train the proposed Morpho-VAE are as follows:

1. Reconstruction Loss (*E*_*Rec*_): binary cross entropy between the input and output images, expressed as ${E}_{Rec}({{{\bf{p}}}},{{{\bf{q}}}})=-1/\dim {{{\bf{p}}}}\mathop{\sum }\nolimits_{i}^{\dim {{{\bf{p}}}}}({p}_{i}\log ({q}_{i})-(1-{p}_{i})\log (1-{q}_{i}))$, where **p** and **q** are the input and output image vectors, respectively.
2. Regularization Loss (*E*_*Reg*_): Kullback–Leibler divergence *D*_*KL*_(*q*(*ζ*∣*X*)∥*p*(*ζ*)) between the data distribution in the latent space *q*(*ζ*∣*X*) encoded by the encoder from data *X* and the predefined reference distribution $p(\zeta )={{{\mathcal{N}}}}(0,1)$, which is fixed as a Gaussian distribution with mean 0 and variance 1.
3. Classification Loss (*E*_*C*_): cross entropy between the predicted ${{{{\bf{y}}}}}^{{\prime} }$ and true label vectors **y** from the latent variable *ζ* and classifier module, expressed as ${E}_{C}=-1/7\mathop{\sum }\nolimits_{i}^{7}{y}_{i}\log ({y}_{i}^{{\prime} })$.

From these three loss functions, VAE loss function is defined as *E*_*VAE*_ = *E*_*Rec*_ + *E*_*Reg*_. Moreover, the total loss function is defined as *E*_*total*_ = (1 − *α*)*E*_*VAE*_ + *αE*_*C*_, where *α* = 0.1 is selected by cross-validation (Fig. 1c), and Morpho-VAE is trained to minimize *E*_*total*_ by backpropagation.

### Hyperparameter tuning

The structural hyperparameters of Morpho-VAE, such as the number of layers, number of filters in each layer, type of activation function, and type of optimization function, were tuned using Optuna^95^.

The number of layers was optimized to be within the range of 1–5, and the number of filters in each layer was optimized to be within the range of 16–128. The activation functions were selected from ReLU, sigmoid, and tanh, and the optimization function was selected from stochastic gradient descent, adaptive momentum estimation (Adam), and RMSprop. Note that the latent-space dimension was fixed to three in these processes. These optimizations were performed by searching 500 different conditions, each with 100 epochs of training, and the following parameters were defined as the optimal hyperparameters to minimize the loss function *E*_*total*_. The other hyperparameters are listed in Supplementary Table 2. The number of layers in the encoder was five. The numbers of filters in each layer were 128, 128, 32, 32, and 64 in the order from the layer nearest to the input layer. The selected activation and optimization functions were ReLU and RMSprop, respectively. Moreover, the number of layers in the decoder was five, and the numbers of filters in each layer were 64, 32, 32, 128, and 128 in the order from the layer nearest to the latent variable. The type of optimization function was RMSprop. Note that sigmoid is adopted instead of ReLU as the activation function of the decoder because the input image of this model is a binary image in the range of [0,1], and the output image needs to be in the same range.

After tuning the structural hyperparameters, the dimensions of the latent variable *ζ* were also explored. The number of dimensions of the latent variable was examined from 2–10 by 100-times independent 100-epoch training with different training–validation datasets for each dimension. Supplementary Fig. 2a illustrates that the mean and median of the minimum of *E*_*total*_ in each 100-epoch training decrease as the dimension increases from two to eight. Because our aim is to select a low-dimensional feature *ζ* that generates a low *E*_*total*_, the dimension value of three was adopted, for which only a slight increase appears in the loss value compared with the dimensions ≥4, but a certain drop (Supplementary Fig. 2a, b) is observed between dimensions two and three.

A double cross-validation procedure^96^ was used for separating the data into training, validation, and test data. One-third of the total data were used as test data to evaluate the generalization performance of Morpho-VAE. Of the remaining data, 75% was separated as training data for tuning the hyperparameters of Morpho-VAE and the remaining 25% as validation data for verifying the hyperparameters to avoid leaks of the same species of data. Because the data set collected in this study had a class imbalance, as listed in Supplementary Table 1, the data set was divided into training and test data using the proportional extraction method, which divides the data by reflecting the sample size of each label. However, due to the limited size of our dataset and the uneven distribution of sexes and species ratios in some of the collected data, it was not feasible to achieve an equal split of sexes or species in the train/validation/test data. Note that two datasets were obtained from one mandible sample (see Datasets section), but the data are distributed such that the same sample is not included both in the test and training data.

### Visualization of the saliency map (Fig. 4a) by Score-CAM

The Score-CAM^51^ method was applied to visualize Morpho-VAE making its decisions. The schematic overview of Score-CAM is presented in Supplementary Fig. 4. First, upsampling is performed from the 8 × 8 pixel activation map, which activates the last layer in the convolution layers of the encoder, to a 128 × 128-pixel image and then normalization is implemented such that the maximum and minimum pixel intensities of the image are 1 and 0, respectively. Each pixel intensity of the image is then multiplied by the intensity of the corresponding pixel in the 128 × 128-pixel original input image to create a masking image. Furthermore, this masking image is re-input into Morpho-VAE and the prediction probability is calculated for the label of the input image through the classifier module. Because the calculated prediction probability can be interpreted as the importance of the masking image, this probability is then multiplied by the activation map, and the final outcome of Score-CAM (Fig. 4a), “the saliency map”, is obtained by taking a sum over the number of filters (e.g., 64).

### Reporting summary

Further information on research design is available in the Nature Research Reporting Summary linked to this article.

## Supplementary information

Supplementary Material

Reporting Summary

## Data Availability

All the data that support this research are provided in the published article, its accompanying Supplementary File, or git https://github.com/masa10223/Morpho-VAE.

## Code availability

The source code used in the study are available at https://github.com/masa10223/Morpho-VAE. All codes are written in Python 3.6, relying on scipy, keras, numpy, pandas, OpenCV, and scikit learn.
