# Supplementary material for: A deep learning approach for morphological feature extraction based on variational auto-encoder: an application to mandible shape
Source: NPJ Syst Biol Appl. 2023 Jul 6;9:30. doi: 10.1038/s41540-023-00293-6 (PMC10322894; doi:10.1038/s41540-023-00293-6)
Supplement: Supplementary file 1 — Supplementary Material [file 41540_2023_293_MOESM1_ESM.pdf]

Supplementary Materials of "A deep learning approach for morphological feature extraction based on variational auto-encoder : an application to mandible shape"

Masato Tsutsumi[1], Nen Saito[2,3,4 *], Daisuke Koyabu[5,6], Chikara Furusawa[1,4,7 *]

[1]Graduate School of Sciences, The University of Tokyo, 7-3-1 Hongo, Tokyo 113-0033, Japan

[2]Graduate School of Integrated Sciences for Life, Hiroshima University, 1-3-1 Kagamiyama, Higashi-Hiroshima City, Hiroshima 739-8528, Japan

[3]Exploratory Research Center on Life and Living Systems, National Institutes of Natural Sciences, 5-1 Higashiyama, Myodaiji-cho, Okazaki, Aichi 444-8787, Japan

[4]Universal Biology Institute, The University of Tokyo, 7-3-1 Hongo, Tokyo 113-0033, Japan

[5]Research and Development Center for Precision Medicine, University of Tsukuba, 1-2 Kasuga, Tsukuba 305-8550, Japan

[6]Jockey Club College of Veterinary Medicine and Life Sciences, City University of Hong Kong, To Yuen Building, Tat Chee Avenue, Kowloon 999077, Hong Kong

[7]Center for Biosystems Dynamics Research, RIKEN, 6-2-3 Furuedai, Suita, Osaka 565-0874, Japan

SUPPLEMENTARY FIGURES

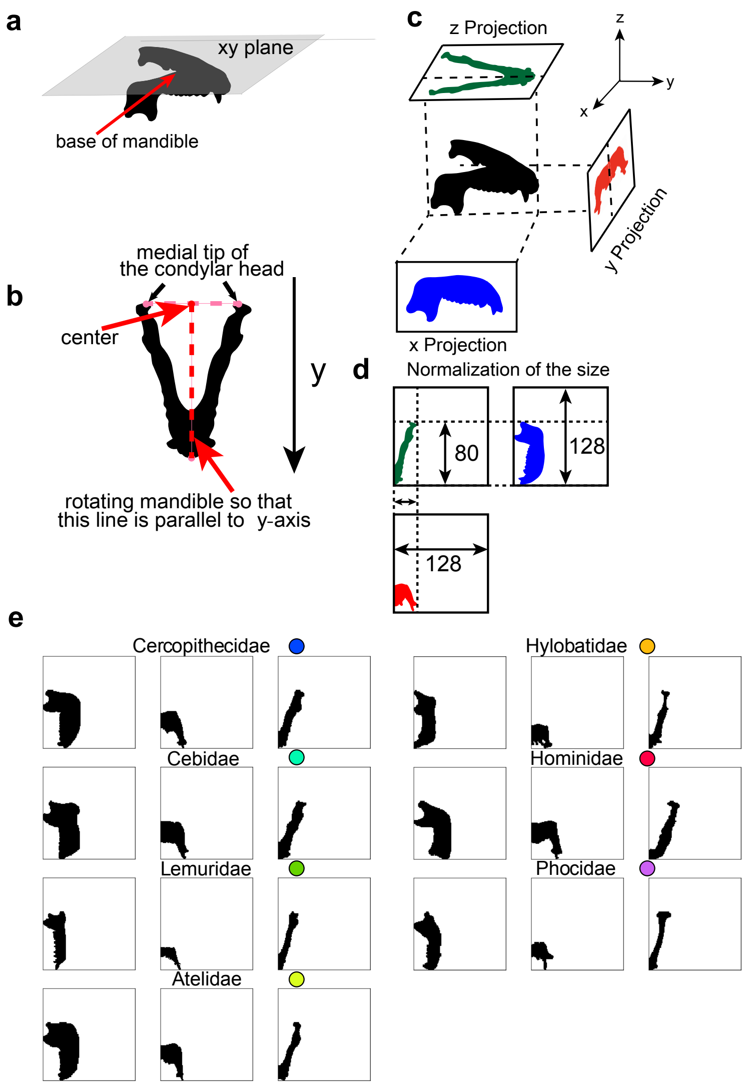

Supplementary Figure 1. Detailed description of image preprocessing.

(A) The xy plane is defined as the plane to which the base of the mandible is horizontal. (B) After placing the mandible in the xy plane, it is rotated so that the mandible tip and the mid-point of the line connecting the condylar head's left and right medial tips have the same y-coordinate. (C) The mandible (arranged as shown) is projected from three orthogonal directions. (D) Normalization of the image. Each projected image is parallel shifted so that the leftmost of the image is tangent to the left edge of the square image frame. Then, the projected images are downsized so that the length from the angular process to the tip of the mandible is 80 pixel. (E) Examples of projected mandible images for each family.

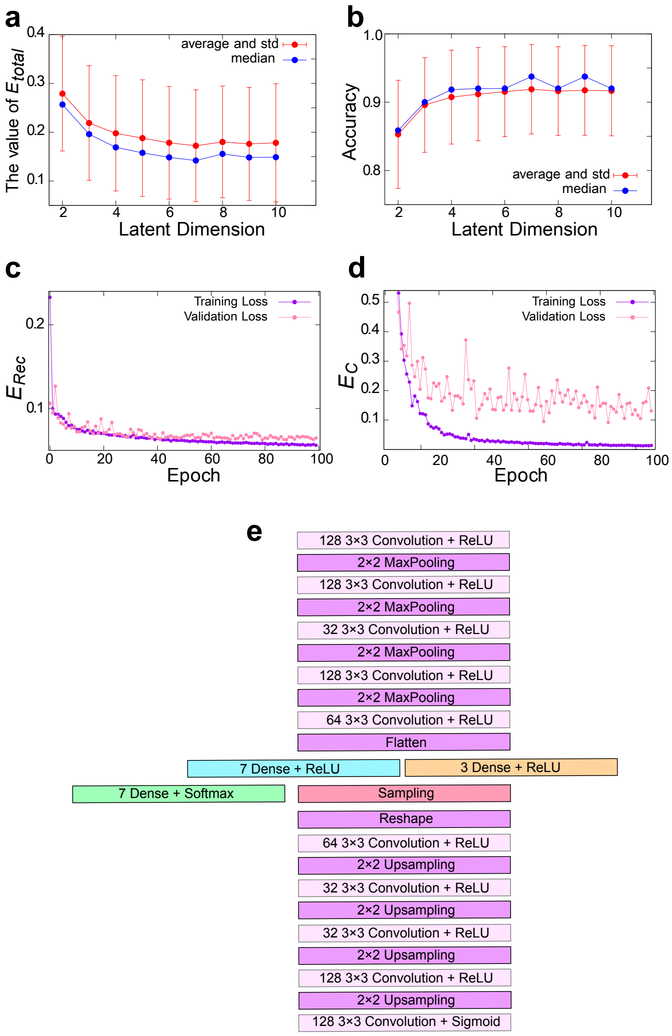

Supplementary Figure 2. Latent dimension tuning and detailed architecture of the proposed Morpho-VAE

(**a**) Mean, standard deviation, and median values of E_{total} for validation data in 2 – 10 dimensions. These values are calculated from 10 independent architectures listed in Table S2. (**b**) Classification accuracy for test data in 2 – 10 dimensions calculated for in 10 independent architectures. The selected number of dimensions is 3. (**c** and **d**) Trajectories of training and validation losses for E_{Rec} and E_C during training. (**e**) Detailed architecture of Morpho-VAE: the upper and lower sides of the figure are the input and output layers, respectively.

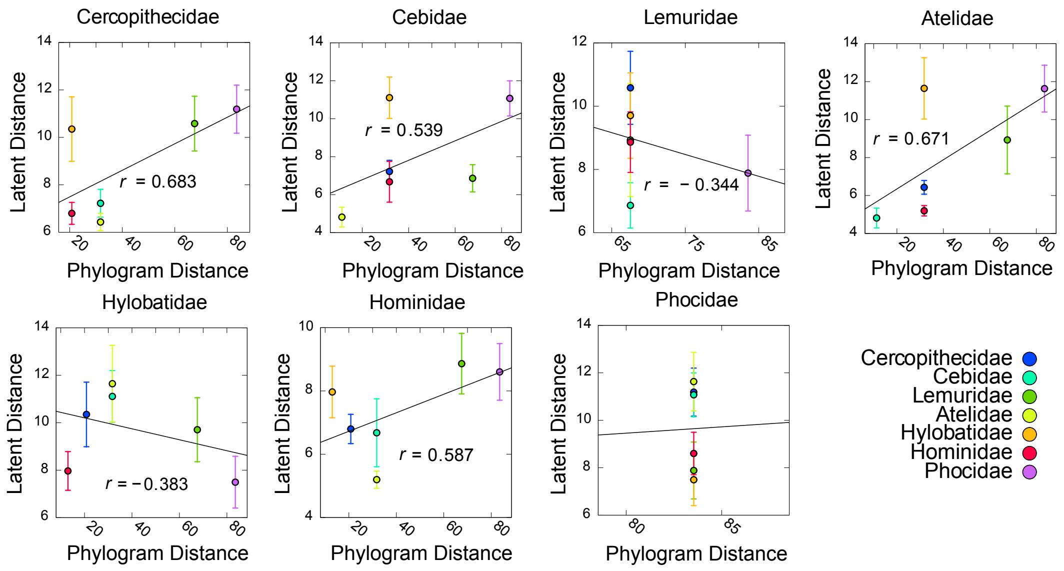

Supplementary Figure 3. **Correlation between the distance in Morpho-VAE and the phylogenetic tree.**

Euclidean distances between family clusters in the latent space of Morpho-VAE are plotted against the age (Ma) of the common ancestor between two families. The error bars indicate standard deviations from the mean calculated from the 10 tuned models listed in Table S2.

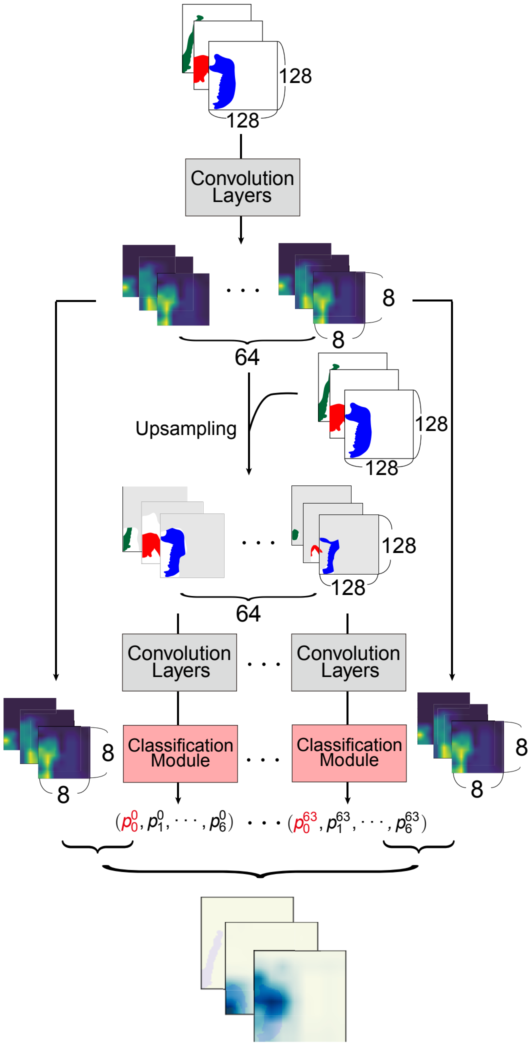

Supplementary Figure 4. **Schematic for creating a saliency map.**

First, the output images with 64 channels obtained from the last convolution layer of Morpho-VAE with a set of input images are prepared. Then, output images are upsampled to the same size as that of the input images. The resulting 64 outputs are normalized using the maximum and minimum values for each image. These 64 normalized output images are multiplied by the original input images to create 64 images. Each of these is then input to Morpho-VAE to calculate the probability of the seven classes. Each of the 64 probability values is considered as the importance of the 64 outputs. Next, the importance and output are multiplied and then added together to obtain a saliency map.

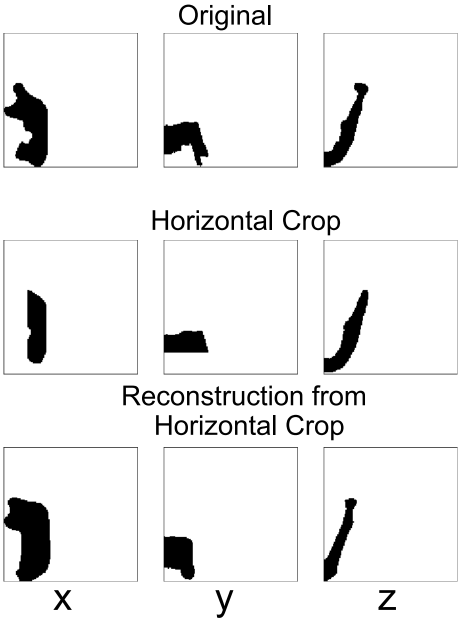

Supplementary Figure 5. **Example of reconstruction failure when crop rate is significantly large.**

A significant defect in the coronoid process results in the failure of the reconstruction, where the reconstructed image is far different from the original image.

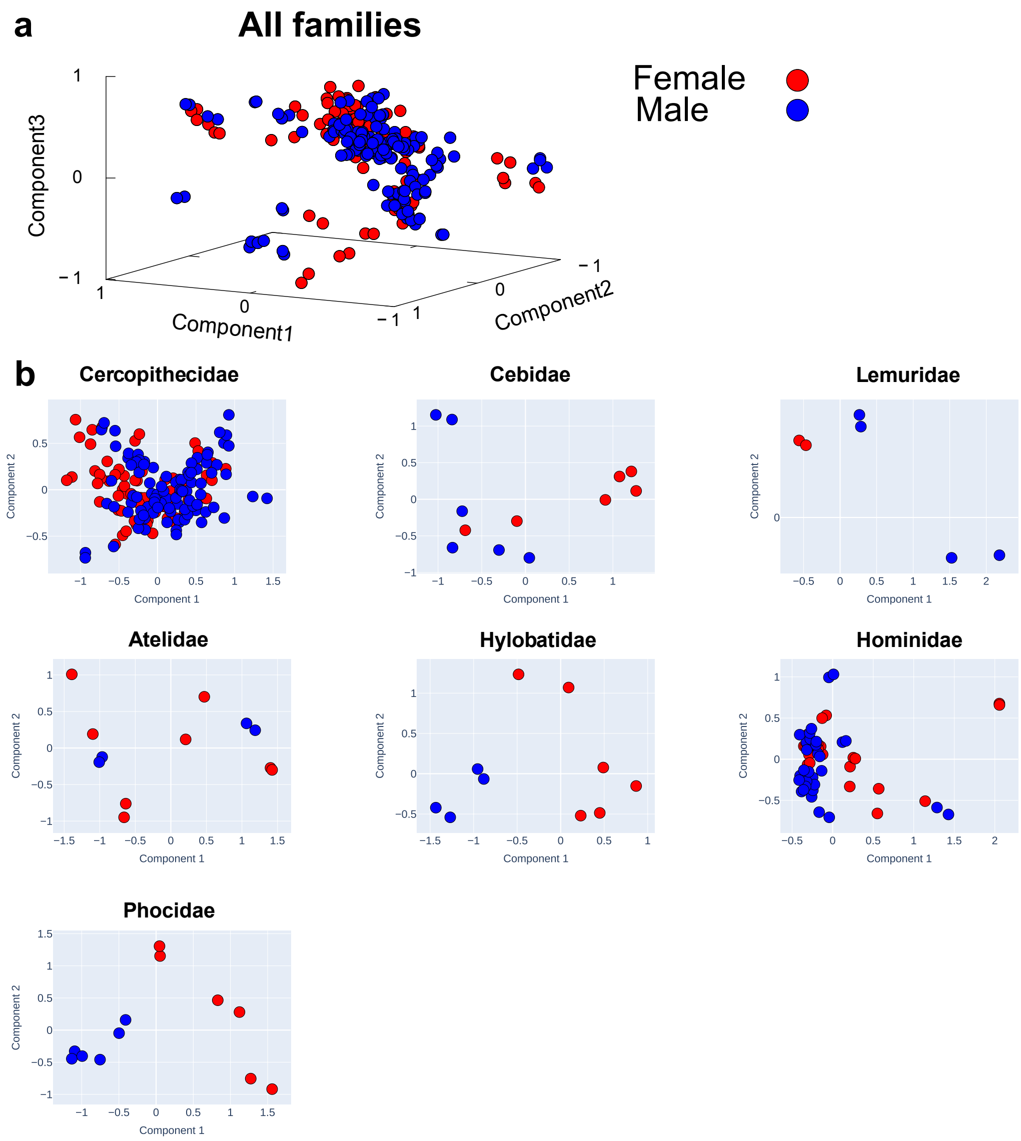

Supplementary Figure 6. Visualization of clustering by sex in each species.

(**a**) The three-dimensional scatter plot for showing how data points with different sexes are distributed in the Morpho-VAE space. (**b**) Two-dimensional plots for each family data. For the visualization, we performed PCA of the three-dimensional latent space of Morpho-VAE and plotted using the top two components. Note that the sexes of unknown sexes are not shown here.

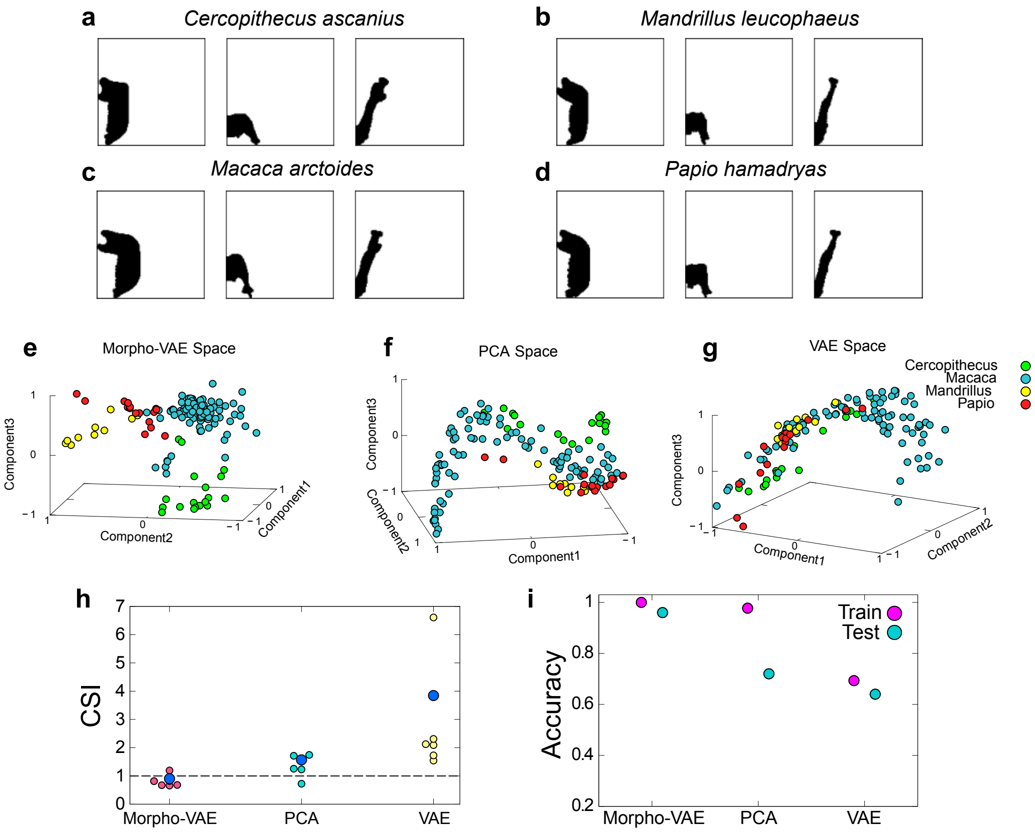

Supplementary Figure 7. **Distribution of data in latent space in the analysis at genus level.**

(**a–d**) Projected images of species belonging to the taxonomic groups Cercopithecus, Macaca, Mandrillus, and Papio. (E-G) Data distribution in latent space: By using Morpho-VAE, PCA, and VAE, respectively, the same input images are compressed into three-dimensional latent space using each method. (H) Dot plot of CSI. Points below 1 represent a pair of well-separated clusters: Blue dots represent Davies-Bouldin indices for different models. (I) The boxplot of the classification accuracy of families using SVM as a cluster separation measure. The classification accuracy was calculated using the best scores in 10 independent runs for each model. Morpho-VAE shows a trend toward higher classification accuracy than PCA and VAE.

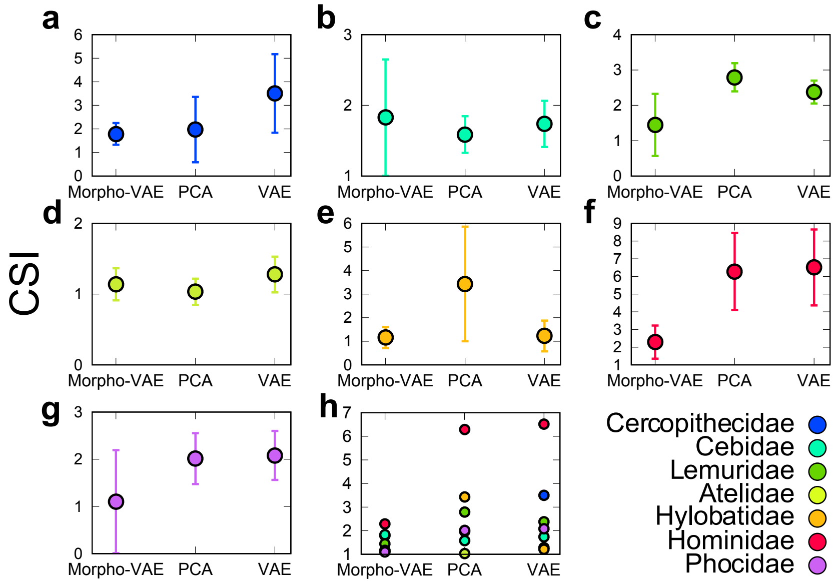

Supplementary Figure 8. Cluster separation index (CSI) for an additional family dataset not included in latent space construction.

(**a–g**) CSI for an additional family not included in the construction of the latent space. The latent spaces were constructed using training data from six out of the seven families. CSI was calculated between the additional family dataset and each of the six families on the latent spaces. The maximum CSI values between the additional family dataset and each of the pre-existing six families are plotted. Each plot represents the mean of the maximum CSI values from 10 different combinations of train and test data. The error bars indicate the standard deviations. (H) Average of the maximum CSI values for all trials shown in A-G.

SUPPLEMENTARY TABLES

Family	Species	Sex	Reference
Cercopethecidae	*Allenopithecus nigroviridis*	M	MorphoSource: ark:/87602/m4/M12503
Cercopethecidae	*Allenopithecus nigroviridis*	M	MorphoSource: ark:/87602/m4/M22953
Cercopethecidae	*Allenopithecus nigroviridis*	M	MorphoSource: ark:/87602/m4/M19904
Cercopethecidae	*Allenopithecus nigroviridis*	M	MorphoSource: ark:/87602/m4/M19465
Cercopethecidae	*Cercocebus galeritus*	M	MorphoSource: ark:/87602/m4/M16154
Cercopethecidae	*Cercopithecus ascanius*	F	MorphoSource: ark:/87602/m4/M23526
Cercopethecidae	*Cercopithecus ascanius*	M	MorphoSource: ark:/87602/m4/M23522
Cercopethecidae	*Cercopithecus ascanius*	M	MorphoSource: ark:/87602/m4/M22476
Cercopethecidae	*Cercopithecus cephus*	F	MorphoSource: ark:/87602/m4/M23554
Cercopethecidae	*Cercopithecus cephus*	M	MorphoSource: ark:/87602/m4/M23550
Cercopethecidae	*Cercopithecus mitis*	M	MorphoSource: ark:/87602/m4/M22386
Cercopethecidae	*Cercopithecus mona*	M	MorphoSource: ark:/87602/m4/M22412
Cercopethecidae	*Cercopithecus neglectus*	M	MorphoSource: ark:/87602/m4/M23530
Cercopethecidae	*Cercopithecus nictitans*	M	MorphoSource: ark:/87602/m4/M23538
Cercopethecidae	*Cercopithecus petaurista*	M	KUPRI: PRICT-1062
Cercopethecidae	*Chlorocebus aethiops*	M	MorphoSource: ark:/87602/m4/M23841
Cercopethecidae	*Colobus angolensis*	F	MorphoSource: ark:/87602/m4/M23519
Cercopethecidae	*Colobus angolensis*	M	MorphoSource: ark:/87602/m4/M22389
Cercopethecidae	*Lophocebus albigena*	M	MorphoSource: ark:/87602/m4/M23834
Cercopethecidae	*Lophocebus albigena*	M	MorphoSource: ark:/87602/m4/M23535
Cercopethecidae	*Macaca arctoides*	F	KUPRI: PRICT-1141
Cercopethecidae	*Macaca arctoides*	F	KUPRI: PRICT-1139
Cercopethecidae	*Macaca arctoides*	F	MorphoSource: ark:/87602/m4/M19915
Cercopethecidae	*Macaca arctoides*	M	MorphoSource: ark:/87602/m4/M19921

**Family**	**Species**	**Sex**	**Reference**
Cercopethecidae	*Macaca assamensis*	F	MorphoSource: ark:/87602/m4/M12494
Cercopethecidae	*Macaca assamensis*	M	MorphoSource: ark:/87602/m4/M19926
Cercopethecidae	*Macaca cyclopis*	M	MorphoSource: ark:/87602/m4/M20097
Cercopethecidae	*Macaca cyclopis*	M	MorphoSource: ark:/87602/m4/M20095
Cercopethecidae	*Macaca fascicularis*	F	MorphoSource: ark:/87602/m4/M19475
Cercopethecidae	*Macaca fascicularis*	M	MorphoSource: ark:/87602/m4/M19481
Cercopethecidae	*Macaca fascicularis*	F	MorphoSource: ark:/87602/m4/M19910
Cercopethecidae	*Macaca fuscata*	F	KUPRI: PRICT-994
Cercopethecidae	*Macaca fuscata*	F	KUPRI: PRICT-998
Cercopethecidae	*Macaca fuscata*	F	KUPRI: PRICT-1242
Cercopethecidae	*Macaca fuscata*	F	KUPRI: PRICT-1256
Cercopethecidae	*Macaca fuscata*	F	KUPRI: PRICT-1260
Cercopethecidae	*Macaca fuscata*	F	KUPRI: PRICT-1262
Cercopethecidae	*Macaca fuscata*	F	KUPRI: PRICT-1264
Cercopethecidae	*Macaca fuscata*	F	KUPRI: PRICT-1266
Cercopethecidae	*Macaca fuscata*	F	KUPRI: PRICT-1268
Cercopethecidae	*Macaca fuscata*	F	KUPRI: PRICT-1270
Cercopethecidae	*Macaca fuscata*	F	KUPRI: PRICT-1272
Cercopethecidae	*Macaca fuscata*	M	KUPRI: PRICT-990
Cercopethecidae	*Macaca fuscata*	M	KUPRI: PRICT-1006
Cercopethecidae	*Macaca fuscata*	M	KUPRI: PRICT-1008
Cercopethecidae	*Macaca fuscata*	M	KUPRI: PRICT-1009
Cercopethecidae	*Macaca fuscata*	M	KUPRI: PRICT-1238
Cercopethecidae	*Macaca fuscata*	M	KUPRI: PRICT-1244
Cercopethecidae	*Macaca fuscata*	M	KUPRI: PRICT-1252
Cercopethecidae	*Macaca fuscata*	M	KUPRI: PRICT-1254
Cercopethecidae	*Macaca maura*	F	MorphoSource: ark:/87602/m4/M20054
Cercopethecidae	*Macaca maura*	M	MorphoSource: ark:/87602/m4/M20057
Cercopethecidae	*Macaca maura*	M	MorphoSource: ark:/87602/m4/M20061

**Family**	**Species**	**Sex**	**Reference**
Cercopethecidae	*Macaca mulatta*	F	MorphoSource: ark:/87602/m4/M19932
Cercopethecidae	*Macaca nemestrina*	F	MorphoSource: ark:/87602/m4/M19473
Cercopethecidae	*Macaca nemestrina*	F	MorphoSource: ark:/87602/m4/M19479
Cercopethecidae	*Macaca nemestrina*	M	MorphoSource: ark:/87602/m4/M19471
Cercopethecidae	*Macaca nemestrina*	M	MorphoSource: ark:/87602/m4/M19477
Cercopethecidae	*Macaca nigra*	F	MorphoSource: ark:/87602/m4/M20128
Cercopethecidae	*Macaca silenus*	M	KUPRI: PRICT-1133
Cercopethecidae	*Macaca sylvanus*	F	KUPRI: PRICT-1135
Cercopethecidae	*Macaca thibetana*	F	MorphoSource: ark:/87602/m4/M19460
Cercopethecidae	*Macaca tonkeana*	F	MorphoSource: ark:/87602/m4/M19986
Cercopethecidae	*Macaca tonkeana*	F	MorphoSource: ark:/87602/m4/M19999
Cercopethecidae	*Macaca tonkeana*	F	MorphoSource: ark:/87602/m4/M20114
Cercopethecidae	*Macaca tonkeana*	M	MorphoSource: ark:/87602/m4/M20005
Cercopethecidae	*Mandrillus leucophaeus*	M	MorphoSource: doi:10.17602/M2/M3060
Cercopethecidae	*Mandrillus leucophaeus*	M	MorphoSource: doi:10.17602/M2/M3063
Cercopethecidae	*Mandrillus leucophaeus*	M	MorphoSource: doi:10.17602/M2/M3074
Cercopethecidae	*Mandrillus leucophaeus*	M	MorphoSource: doi:10.17602/M2/M3100
Cercopethecidae	*Mandrillus sphinx*	F	MorphoSource: ark:/87602/m4/M19468
Cercopethecidae	*Papio hamadryas*	F	MorphoSource: ark:/87602/m4/M19438
Cercopethecidae	*Papio hamadryas*	F	MorphoSource: ark:/87602/m4/M19455
Cercopethecidae	*Papio hamadryas*	F	MorphoSource: ark:/87602/m4/M23866
Cercopethecidae	*Papio hamadryas*	M	MorphoSource: ark:/87602/m4/M20063
Cercopethecidae	*Papio hamadryas*	M	MorphoSource: ark:/87602/m4/M22421
Cercopethecidae	*Papio hamadryas*	M	MorphoSource: ark:/87602/m4/M22431
Cercopethecidae	*Papio hamadryas*	M	MorphoSource: ark:/87602/m4/M23872
Cercopethecidae	*Papio hamadryas*	M	MorphoSource: ark:/87602/m4/M23877
Cercopethecidae	*Presbytis femoralis*	F	KUPRI: PRICT-1053
Cercopethecidae	*Presbytis femoralis*	M	KUPRI: PRICT-1051
Cercopethecidae	*Presbytis melalophos*	F	KUPRI: PRICT-1054

**Family**	**Species**	**Sex**	**Reference**
Cercopethecidae	*Presbytis melalophos*	M	MorphoSource: ark:/87602/m4/M22472
Cercopethecidae	*Simias concolor*	?	KUPRI: PRICT-1094
Cercopethecidae	*Simias concolor*	?	KUPRI: PRICT-1098
Cercopethecidae	*Theropithecus gelada*	M	MorphoSource: ark:/87602/m4/M13684
Cercopethecidae	*Theropithecus gelada*	M	MorphoSource: ark:/87602/m4/M19458
Cebidae	*Cebus albifrons*	M	MCPA2: DKY0100
Cebidae	*Cebus apella*	F	KUPRI: PRICT-1066
Cebidae	*Cebus apella*	F	KUPRI: PRICT-1069
Cebidae	*Cebus apella*	M	MCPA2: DKY2326
Cebidae	*Cebus capucinus*	F	MCPA2: DKY2632
Cebidae	*Cebus capucinus*	M	MCPA2: DKY1811
Lemuridae	*Hapalemur griseus*	M	MorphoSource: doi:10.17602/M2/M2710
Lemuridae	*Hapalemur griseus*	M	MorphoSource: ark:/87602/m4/M13091
Lemuridae	*Lemur catta*	F	MCPA2: DKY2715
Lemuridae	*Lemur catta*	M	MCPA2: DKY2436
Lemuridae	*Lemur catta*	?	MorphoSource: doi:10.17602/M2/M2941
Lemuridae	*Lemur catta*	?	MorphoSource: ark:/87602/m4/M13091
Atelidae	*Alouatta caraya*	F	MorphoSource: doi:10.17602/M2/M2854
Atelidae	*Ateles fusciceps*	F	MorphoSource ark:/87602/m4/85533
Atelidae	*Ateles fusciceps*	M	MorphoSource ark:/87602/m4/87860
Atelidae	*Ateles geoffroyi*	F	MorphoSource: ark:/87602/m4/350987
Atelidae	*Ateles paniscus*	F	MCPA2: DKY0649
Atelidae	*Ateles paniscus*	M	MCPA2: DKY1073
Hylobatidae	*Hylobates hoolock*	F	MCPA2: DKY4057
Hylobatidae	*Hylobates hoolock*	?	MCPA2: DKY2364
Hylobatidae	*Hylobates lar*	F	MCPA2: DKY1267
Hylobatidae	*Hylobates lar*	F	MCPA2: DKY1696
Hylobatidae	*Hylobates syndactylus*	F	MCPA2: DKY1452
Hylobatidae	*Hylobates syndactylus*	M	MCPA2: DKY3268

**Family**	**Species**	**Sex**	**Reference**
Homonidae	*Gorilla gorilla*	F	KUPRI
Homonidae	*Gorilla gorilla*	F	KUPRI
Homonidae	*Gorilla gorilla*	F	KUPRI
Homonidae	*Gorilla gorilla*	?	KUPRI
Homonidae	*Gorilla gorilla*	M	MCPA2: DKY1755
Homonidae	*Homo sapiens*	F	MCPA2: DKY9004
Homonidae	*Homo sapiens*	F	MorphoSource: ark:/87602/m4/394379
Homonidae	*Homo sapiens*	F	MorphoSource: ark:/87602/m4/394469
Homonidae	*Homo sapiens*	F	MorphoSource: ark:/87602/m4/394426
Homonidae	*Homo sapiens*	F	MorphoSource: ark:/87602/m4/395136
Homonidae	*Homo sapiens*	F	MorphoSource: ark:/87602/m4/395262
Homonidae	*Homo sapiens*	F	MorphoSource: ark:/87602/m4/395280
Homonidae	*Homo sapiens*	M	MorphoSource: ark:/87602/m4/397135
Homonidae	*Homo sapiens*	M	MorphoSource: ark:/87602/m4/394299
Homonidae	*Homo sapiens*	M	MorphoSource: ark:/87602/m4/394385
Homonidae	*Homo sapiens*	M	MorphoSource: ark:/87602/m4/394210
Homonidae	*Homo sapiens*	M	MorphoSource: ark:/87602/m4/395180
Homonidae	*Homo sapiens*	M	MorphoSource: ark:/87602/m4/395171
Homonidae	*Homo sapiens*	M	MorphoSource: ark:/87602/m4/394435
Homonidae	*Homo sapiens*	M	MorphoSource: ark:/87602/m4/394330
Homonidae	*Homo sapiens*	M	MorphoSource: ark:/87602/m4/394454
Homonidae	*Homo sapiens*	M	MorphoSource: ark:/87602/m4/395117
Homonidae	*Homo sapiens*	M	MorphoSource: ark:/87602/m4/395156
Homonidae	*Homo sapiens*	M	MorphoSource: ark:/87602/m4/395239
Homonidae	*Homo sapiens*	M	MorphoSource: ark:/87602/m4/395271
Homonidae	*Homo sapiens*	M	MorphoSource: ark:/87602/m4/394444
Homonidae	*Pan troglodytes*	F	MCPA2: DKY2763
Homonidae	*Pan troglodytes*	M	MCPA2: DKY0218
Homonidae	*Pongo pygmaeus*	F	MCPA2: DKY4095

Family	Species	Sex	Reference
Homonidae	*Pongo pygmaeus*	M	MCPA2: DKY5011
Phocidae	*Zalophus californianus*	F	MorphoSource: ark:/87602/m4/M75750
Phocidae	*Zalophus californianus*	F	MorphoSource: ark:/87602/m4/M75755
Phocidae	*Zalophus californianus*	F	MorphoSource: ark:/87602/m4/M75757
Phocidae	*Zalophus californianus*	M	MorphoSource: ark:/87602/m4/M75753
Phocidae	*Zalophus californianus*	M	MorphoSource: ark:/87602/m4/M75759
Phocidae	*Zalophus californianus*	M	MorphoSource: ark:/87602/m4/M75761

Supplemental Table 1. The mandible data is presented in the following format: family, species name, sex, and data source. The data sources include MorphoSource (available at https://www.morphosource.org/), Kyoto University Primate Research Institute (KUPRI), and Mammalian Crania Photographic Archive Second Edition (MCPA2), with each followed by the respective DOI or ID number.

# of layers	# of filters in 1st layer	# of filters in 2nd layer	# of filters in 3rd layer	# of filters in 4th layer	# of filters in 5th layer	Activation function	Optimization function
5	64	80	64	64	16	ReLU	Adam
5	112	128	128	32	32	tanh	Adam
5	96	64	112	128	96	ReLU	RMSprop
5	16	64	16	96	112	ReLU	Adam
5	112	16	112	128	16	ReLU	RMSprop
5	64	80	64	112	48	ReLU	RMSprop
5	80	16	32	80	64	ReLU	RMSprop
5	128	128	32	32	64	ReLU	RMSprop
5	32	48	48	48	32	ReLU	RMSprop
5	48	64	64	64	112	ReLU	RMSprop

Supplemental Table 2. All model hyperparameters. 10 independent hyperparameters that are obtained through hyperparameter optimization are listed. Adam represents adaptive moment estimation, while RMSprop indicates root mean square propagation.
